# Leukocyte telomere length, allelic variations in related genes and risk of coronary heart disease in people with long-standing type 1 diabetes

**DOI:** 10.1186/s12933-022-01635-0

**Published:** 2022-10-11

**Authors:** Manuel Sanchez, Caroline Kannengiesser, Sophie Hoang, Louis Potier, Frédéric Fumeron, Nicolas Venteclef, André Scheen, Jean-François Gautier, Samy Hadjadj, Michel Marre, Ronan Roussel, Kamel Mohammedi, Gilberto Velho

**Affiliations:** 1 INSERM UMR-S1151, CNRS UMR-S8253, Institut Necker-Enfants Malades, Université Paris Cité, Paris, France; 2 UFR de Médecine, Université Paris Cité, Paris, France; 3 Department of Geriatrics, Assistance Publique - Hôpitaux de Paris, Bichat University Hospital, 46 rue Henri Huchard, 75018 Paris, France; 4 Department of Genetics, Assistance Publique - Hôpitaux de Paris, DHU FIRE, Bichat Hospital, Paris, France; 5 Department of Geriatrics, Charles-Foix University Hospital, Vitry sur Seine, France; 6 Department of Diabetology, Endocrinology and Nutrition, Assistance Publique - Hôpitaux de Paris, DHU FIRE, Bichat Hospital, Paris, France; 7 Department of Diabetology, Endocrinology and Nutrition, Sart Tilman University Hospital, Liège, Belgium; 8 Department of Diabetology, Endocrinology and Nutrition, Assistance Publique - Hôpitaux de Paris, Lariboisière University Hospital, Paris, France; 9 Institut du Thorax, INSERM, CNRS, CHU Nantes, Université de Nantes, Nantes, France; 10 Clinique Ambroise Paré, Neuilly-sur-Seine, France; 11 INSERM U1034, Bordeaux University and Hospital, Bordeaux, France

**Keywords:** Telomere, Leukocyte telomere length, Coronary heart disease, Type 1 diabetes, Cohort study

## Abstract

**Background:**

Type 1 diabetes is associated with accelerated vascular aging and advanced atherosclerosis resulting in increased rates of cardiovascular disease and premature death. We evaluated associations between Leukocyte telomere length (LTL), allelic variations (SNPs) in LTL-related genes and the incidence of coronary heart disease (CHD) in adults with long-standing type 1 diabetes.

**Methods:**

We assessed associations of LTL, measured at baseline by RT–PCR, and of SNPs in 11 LTL-related genes with the risk of coronary heart disease (CHD: myocardial infarction or coronary revascularization) and all-cause death during follow-up in two multicenter French-Belgian prospective cohorts of people with long-standing type 1 diabetes.

**Results:**

In logistic and Cox analyses, the lowest tertile of LTL distribution (short telomeres) at baseline was associated with the prevalence of myocardial infarction at baseline and with increased risk of CHD (Hazard ratio 3.14 (1.39–7.70), p = 0.005, for shorter vs longer tertile of LTL) and all-cause death (Hazard ratio 1.63 (95% CI 1.04–2.55), p = 0.03, for shorter vs combined intermediate and longer tertiles of LTL) during follow-up. Allelic variations in six genes related to telomere biology (TERC, NAF1, TERT, TNKS, MEN1 and BICD1) were also associated with the incidence of CHD during follow-up. The associations were independent of sex, age, duration of diabetes, and a range of relevant confounding factors at baseline.

**Conclusions:**

Our results suggest that short LTL is an independent risk factor for CHD in people with type 1 diabetes.

**Supplementary Information:**

The online version contains supplementary material available at 10.1186/s12933-022-01635-0.

## Background

Type 1 diabetes is associated with accelerated vascular aging and advanced atherosclerosis resulting in increased rates of cardiovascular disease (CVD) and premature death [1]. Intensive glycemic control was shown to reduce the long-term risk for atherosclerotic cardiovascular complications [2]. However, the risk of cardiovascular death in people with well-controlled type 1 diabetes remains twice as high as in the general population [3]. Understanding the molecular mechanisms involved in the pathogenesis of atherosclerosis in type 1 diabetes is still an important issue to identify new potential therapeutic approaches.

Telomeres are DNA–protein structures that play a major role in the protection of chromosomes against fusion and degradation [4]. Telomere shortening and dysfunction is involved in the development of age-related diseases [5], including vascular senescence [6]. Observational studies have highlighted consistent associations between telomere length and the risk of CVD in the general population [7–9]. Moreover, leukocyte telomere length (LTL) was associated with the risk of coronary heart disease (CHD) in people with type 2 diabetes [10]. LTL was previously associated in people with type 1 diabetes with the progression of chronic kidney disease [11] and with the risk of lower-limb amputation [12], but no data is available regarding CHD.

Genome-wide association studies (GWAS) identified several loci associated with LTL [13], suggesting a genetic susceptibility to LTL variability. In addition to genetic determinants, many modifiable factors, including medications, environment and lifestyle, may affect telomere length throughout lifetime [14, 15]. Telomeres have been proposed as potential therapeutical targets for CVD [15], and thus, telomere therapeutics could have clinical implications in the treatment and care of people with diabetes, particularly of people with type 1 diabetes who will typically have a diagnosis in early life and a lifelong duration of diabetes.

We hypothesized that accelerated telomere shortening may play a role in the pathophysiology of CHD in type 1 diabetes. In the present investigation, we assessed associations of LTL measured at baseline and of allelic variations in LTL-related genes with the risk of coronary events during long-term follow-up in two cohorts of people with long-standing type 1 diabetes.

## Methods

### Study participants

We analyzed data from two French and Belgian prospective cohorts of people with type 1 diabetes. The *Génétique de la Néphropathie Diabétique* (GENEDIAB) study was a multi-center cohort conducted in 17 diabetes clinics in France and Belgium (see list of centers in Additional file 1) [16]. GENEDIAB participants were recruited from May 1994 to April 1995 based on the diagnosis of type 1 diabetes before the age of 35 years, duration of diabetes of at least 5 years, with a past or present history of pre-proliferative or proliferative diabetic retinopathy requiring laser photocoagulation therapy. The GENESIS France-Belgique study was a family-based cohort including probands with type 1 diabetes for at least 5 years [17]. GENESIS participants were recruited from November 1998 to December 2000 on the basis of a diagnosis of type 1 diabetes before the age of 35 years, with initial ketosis and requirement for permanent insulin treatment within 1 year of diagnosis, and past or present diagnosis of diabetic retinopathy. The study protocol was approved by the Ethics Committee of Angers University Hospital (Angers, France), and all participants gave written informed consent.

Participants were followed until death or the latest clinical visit up to May 31, 2019. Clinical and biological data were obtained from hospital case records or by contacting the family physician of participants. Vital status was cross-checked by contacting the civil registry of the birth place of participants. The present investigation was performed in 767 GENEDIAB or GENESIS participants for whom baseline DNA samples and incident CHD data during follow-up were available. The Flow chart of participants to the present study is shown in the Additional file 1: Fig. S1. Clinical characteristics at baseline of GENEDIAB and GENESIS participants selected for the present investigation are shown in the Additional file 1: Table S1. Characteristics of participants selected or not for the present investigation are shown in the Additional file 1: Table S2.

### Clinical outcomes

Incident CHD, the primary outcome, was defined as the occurrence of myocardial infarction or the requirement of coronary revascularization during follow-up, whichever occurred first. Myocardial infarction was diagnosed as the occurrence of at least 2 out of 3 of the following criteria: constrictive chest pain lasting 20 min or longer, increased serum creatinine phosphokinase and/or troponin levels, or typical electrocardiographic changes. Baseline characteristics of participants by the incidence of CHD during follow-up are summarized in Table 1. A secondary outcome defined as the occurrence of death of any cause during follow-up was also investigated.

**Table 1:** Characteristics of participants at baseline by CHD incidence during follow-up: LTL and SNP studies

	**LTL study (GENEDIAB cohort)**			**SNP studies (all subjects)**		
	**Incident CHD**		**p**	**Incident CHD**		**p**
	**No**	**Yes**		**No**	**Yes**	
N (%)	205 (79)	55 (21)	–	648 (84)	119 (16)	–
Sex: male, n (%)	119 (58)	36 (65)	0.36	343 (53)	78 (66)	0.01
LTL, T/S ratio*	1.35 [1.03]	1.18 [0.87]	0.05	–	–	–
Age, y	43 ± 11	50 ± 13	0.0005	42 ± 11	50 ± 12	< 0.0001
Duration of diabetes, y	28 ± 9	32 ± 11	0.004	27 ± 9	32 ± 11	< 0.0001
BMI, kg/m^2^	23.9 ± 3.3	24.4 ± 3.1	0.33	24.1 ± 3.5	25.1 ± 3.8	0.004
Systolic blood pressure, mmHg	138 ± 18	142 ± 18	0.13	134 ± 19	140 ± 18	0.0009
Diastolic blood pressure, mmHg	79 ± 12	80 ± 11	0.69	77 ± 11	79 ± 11	0.06
HbA1c, %	8.5 ± 1.5	8.8 ± 1.7	0.27	8.5 ± 1.5	8.6 ± 1.5	0.43
HbA1c, mmol/mol	69 ± 17	72 ± 18	0.27	70 ± 16	71 ± 16	0.43
Total cholesterol, mmol/l	5.67 ± 1.42	5.66 ± 1.07	0.96	–	–	–
eGFR, ml/min/1.73 m^2^	76 ± 29	68 ± 30	0.07	84 ± 31	73 ± 31	0.001
UAC, mg/l*	36 [457]	39 [258]	0.80	22 [187]	45[361]	0.04
UAC stages: Normoalbuminuria, n (%)	77 (38)	19 (35)		310 (48)	39 (33)	
Microalbuminuria, n (%)	44 (21)	10 (18)	0.69	139 (21)	27 (23)	0.004
Macroalbuminuria, n (%)	84 (41)	26 (47)		199 (31)	53 (44)	
Current tobacco smoking, n (%)	53 (26)	9 (16)	0.17	183 (28)	29 (25)	0.50
Previous myocardial infarction, n (%)	11 (5.4)	7 (12.7)	0.07	17 (2.6)	13 (10.9)	0.0002
Previous stroke, n (%)	6 (2.9)	3 (5.5)	0.40	15 (2.3)	6 (5.1)	0.12
Previous LLA, n (%)	30 (15)	18 (33)	0.005	39 (6.1)	25 (21.2)	< 0.0001
Use of lipid lowering drugs, n (%)	16 (8)	5 (9)	0.78	41 (6)	20 (17)	0.0004
Use of blood pressure lowering drugs, n (%)	112 (55)	36 (69)	0.06	322 (50)	83 (72)	< 0.0001
Use of ACE-I, n (%)	90 (44)	26 (50)	0.44	250 (39)	62 (53)	0.003

Quantitative data expressed as mean ± SD or median [IQR]*. Statistics are Student's *t* test, *BMI* body mass index, *eGFR* estimated glomerular filtration rate, *UAC* urinary albumin concentration, *LLA* lower limb amputation, *ACE-I* angiotensin converting enzyme inhibitor

p < 0.05 was significant

*Kruskal–Wallis test or Fisher's exact test. Incident CHD defined as the occurrence of myocardial infarction or the requirement of coronary revascularization during follow-up. LTL: leukocyte telomere length T/S ratio: Telomere to a single gene (used as a control) ratio (see “Methods” section)

### Measurement of LTL

LTL was measured in triplicate on DNA samples from the GENEDIAB cohort, collected at baseline and kept frozen at − 80 °C. We used relative quantification by Polymerase chain reaction (PCR) adapted from method described by Cawthon in 2002 [18] to determine the relative telomere to single copy gene (T/S) ratio. The DNA of a non-diabetic control was used as a calibrator and measured in every plate to allow the inter-plate comparisons. The primers described in 2009 by Cawthon [19] were used for telomeres amplification:

- TEL-G, CACTAAGGTTTGGGTTTGGGTTTGGGTTTGGGTTAGTGT

- TEL-C, TGTTAGGTATCCCTATCCCTATCCCTATCCCTATCCCTAACA

The TEL-G and TEL-C primers were diluted at 100 and 300 nM concentrations, respectively. The Glyceraldehyde-3-Phosphate-Deshydrogenase (GAPDH) gene was used as the single copy gene. PCR preparation was set up by mixing 1.5 µL of DNA sample at 2.5 ng/µL concentration with 1.5 µL of water, 5 µL of SYBER Green I Master and 1 µL of each type of a couple of primers (forward and reverse). DNA was replaced by water in the negative controls. PCR were assessed on a Lightcycler 480 (Roche LifeScience^®^) using the thermic cycling as follow: after a common activation phase at 95 °C during 10 min, samples were submitted to 35 cycles with 95 °C during 5 s, then 59 °C during 10 s followed by 72 °C during 2 min for telomeres amplification and 95° during 10 s, then 62 °C during 15 s followed by 72 °C during 15 s for the GAPDH gene. According to previous described methods [20, 21], the T/S ratio was estimated after correction of the difference in PCR efficiency (E) between telomere and reference gene amplification as follow: T/S = E(telomere)^ΔCt^ ^telomeres^/E(GAPDH)^ΔCt^ ^GAPDH^. The efficiencies (E) of the two PCR (telomere and reference genes) were estimated using standard curves and the formula E = 10^–1/slope^ and the ΔCt represented the difference between the thresholds (CT) at which the fluorescence was detected by the machine, for the calibrator and the participant samples during telomeres and reference gene amplifications, respectively.

### Selection of SNPs and genotyping assay

We selected 33 SNPs with a minor allele frequency > 5% in 11 genes previously associated with LTL in GWAS or candidate gene studies in the general population of European descent [13, 22–26] (Additional file 1: Table S3): TERT (Telomerase Reverse Transcriptase), TERC (Telomerase RNA Component), STN1 (STN1 Subunit of CST Complex), TERF1 (Telomeric repeat-binding factor 1), NAF1 (Nuclear Assembly Factor 1), TNKS (Tankyrase), MRE11A (Meiotic Recombination 11 homolog A), BICD1 (Bicaudal D homolog 1), MEN1 (Multiple Endocrine Neoplasia type 1), MPHOSPH6 (M-phase Phosphoprotein 6) and ZNF208 (Zinc Finger Protein 208). Genotypes were determined by competitive allele-specific PCR genotyping system assays (KASP, LGC Genomics, Hoddeston, UK). Genotyping success rate ranged from 83 to 96% (mean ± SD: 91 ± 3%). Genotypes were in Hardy–Weinberg equilibrium (Pearson’s chi-squared test with 1 degree of freedom p > 0.01).

### Computations and statistical analyses

Categorical variables were expressed as number of participants with corresponding percentage. Continuous variables were expressed as mean ± SD or as median and interquartile range (IQR) for those with skewed distribution. Two sets of analyses were performed. In a first set we analyzed associations of LTL with a previous history of myocardial infarction at baseline and with outcome incidences during follow-up in 260 GENEDIAB participants for whom LTL and follow-up data were available. In a second set, we analyzed associations of the SNPs with the outcomes in 767 participants from GENEDIAB (n = 323) or GENESIS (n = 444) for whom genotyping and follow-up data were available. Data from the cohorts were pooled to increase sample size and the number of events during follow-up, and thus the statistical power of the SNP analyses. Characteristics of participants at baseline were compared using Pearson's chi-squared test, Fisher’s exact test, Student’s *t* test, ANOVA or Kruskal–Wallis test. Associations of LTL with the prevalence of myocardial infarction at baseline were assessed by logistic regression analyses, adjusted for relevant confounding covariates (see regression model 1 below), with odds ratios (OR) and associated 95% confidence interval (CI) computed for tertiles of LTL distribution and for 1 SD of log[LTL]. For the computations of OR and hazard ratios (HR; see below) for 1 SD of log[LTL], a Z-score of log[LTL] was calculated for each participant taking into account the mean and SD of log[LTL] in the GENEDIAB cohort. Kaplan–Meier curves were used to plot the incidences of outcomes over time, which were compared by log-rank test. Cox proportional hazards regression models were fitted to estimate associations of LTL or SNPs with the outcomes. Hazard Ratios (HR) with associated 95% confidence interval (CI) were computed for the risk allele of the SNPs, and for tertiles of LTL distribution and 1 SD of log[LTL]. OR and HR were adjusted for sex, age, body mass index (BMI), duration of diabetes, tobacco smoking, glycated hemoglobin (HbA1c), estimated glomerular filtration rate (eGFR), urinary albumin concentration (UAC), and use angiotensin converting enzyme inhibitor, antihypertensive and lipid lowering drugs at baseline (Model 1), plus a previous history of myocardial infarction at baseline (Model 2; Cox analyses only). Circulating lipids were available only for GENEDIAB participants. For the sake of consistency between analyses, the use of lipid lowering drugs, available for all participants, was considered as a proxy of circulating lipid status and was included as a covariate in the regression models. For the analyses in pooled cohorts, we verified that genotype and allele frequencies from the SNPs were similar in participants from both cohorts, and cohort membership was included in the regression models to take into account cohort-related differences. For each SNP analysis, a genetic model for the risk allele (dominant, codominant, recessive) was chosen by looking at the frequency of outcome incidence by the SNP genotype. Two sets of sensitivity analyses were performed. First, Cox models were fitted to estimate associations of LTL with new cases of CHD during follow-up, considering only participants without a history of CHD at baseline Second, as death could compete with the occurrence of CHD, we have also performed competing-risk regression analyses according to the Fine and Gray method [27] with death from all cause during follow-up as a competing risk (Model 3). Subhazard ratios (sHR) with 95% CI were computed for tertiles of LTL and for 1 SD of log[LTL]. Statistics were performed with JMP (www.jmp.com) and Stata (www.stata.com) softwares. P < 0.05 was considered significant.

## Results

### LTL and myocardial infarction at baseline

Baseline characteristics by tertiles of LTL distribution are summarized in the Additional file 1: Table S4. The prevalence of previous myocardial infarction at baseline by tertiles of LTL distribution was 12.2% (T1: short LTL), 4.7% (T2: intermediate LTL) and 3.6% (T3: long LTL), respectively (p = 0.04). Cox analyses confirmed the association of short LTL, expressed as tertiles of the distribution or as a continuous variable, with the prevalence of myocardial infarction at baseline (Table 2).

**Table 2:** Myocardial infarction at baseline and CHD risk during follow-up by baseline LTL in the GENEDIAB cohort

	Crude		Adjusted Model 1		Adjusted Model 2	
	OR or HR (95% CI)	p	OR or HR (95% CI)	p	HR (95% CI)	p
Previous MI at baseline*						
T1 vs T3	3.76 (1.12–17.08)	0.03	7.84 (1.65–54.57)	0.008		
T1 vs T2	2.85 (0.93–10.64)	0.07	4.56 (1.15–22.61)	0.03		
T2 vs T3	1.32 (0.28–6.86)	0.72	1.72 (0.28–12.36)	0.55		
Z-score log[LTL]	0.60 (0.36–0.99)	0.05	0.31 (0.14–0.62)	0.0007		
CHD at follow-up						
T1 vs T3	2.55 (1.26–5.57)	0.009	3.06 (1.37–7.43)	0.006	3.14 (1.39–7.70)	0.005
T1 vs T2	1.56 (0.87–2.85)	0.14	1.84 (0.91–3.84)	0.09	1.63 (0.80–3.39)	0.18
T2 vs T3	1.63 (0.78–3.63)	0.20	1.66 (0.72–4.08)	0.24	1.92 (0.81–4.91)	0.14
Z-score log[LTL]	0.76 (0.58– 1.01)	0.06	0.71 (0.52–0.96)	0.03	0.73 (0.54–0.98)	0.03

^*^Odds ratio (OR) computed by logistic regression analyses and Hazard Ratio (HR) computed by Cox proportional hazards survival regression analysis for 1 SD of log[LTL] and for tertiles (T) of LTL distribution. T1 (short LTL), T2 (intermediate LTL), T3 (long LTL). Model 1: adjusted for sex, age, BMI, duration of diabetes, HbA1c, eGFR, UAC, tobacco smoking and use of ACE-Inhibitors, antihypertensive and lipid lowering drugs at baseline. Model 2: Model 1 plus adjustment for previous history of myocardial infarction at baseline. Number of participants with/without a previous myocardial infarction (MI) at baseline by LTL tertiles: 11/79 (T1), 4/82 (T2) and 3/81 (T3). Number of participants with/without incident CHD during follow-up by LTL tertiles: 25/65 (T1), 20/72 (T2) and 10/68 (T3)

### Outcomes during follow-up by baseline LTL

**Fig. 1:**
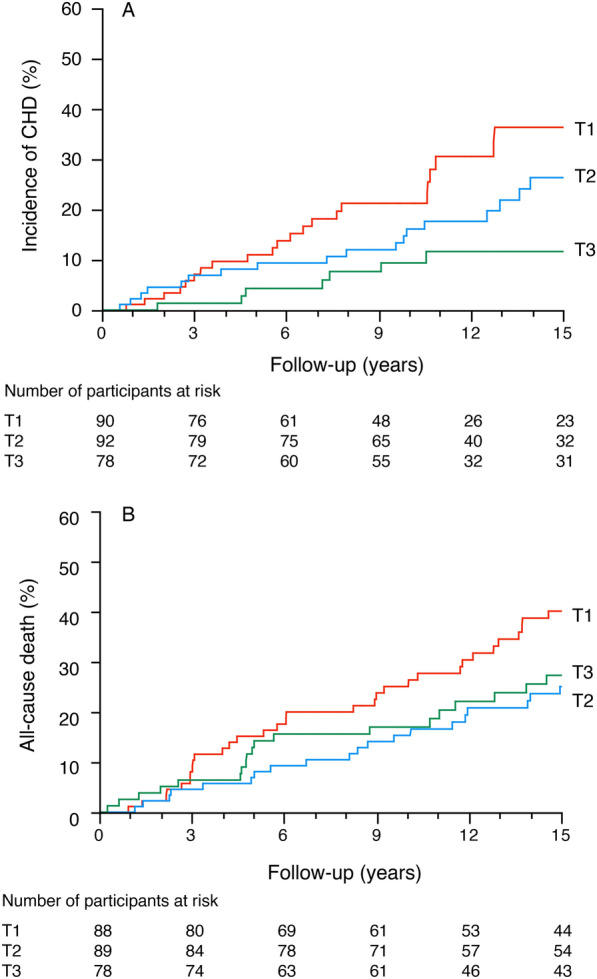
Kaplan–Meier curve for the incidence of outcomes in the GENEDIAB cohort during follow-up by tertiles of LTL distribution at baseline: T1 (short LTL), T2 (intermediate LTL), T3 (long LTL). A) Incidence of CHD. Log-rank test chi square 6.96, p = 0.03. B) All-cause death. Log-rank test chi square 6.75, p = 0.03

The mean ± SD duration of follow-up for the 260 GENEDIAB participants selected for the LTL study was 12 ± 7 years. Myocardial infarction and coronary revascularization occurred in 42 (16%) and 33 (13%) participants during follow-up, with 20 participants presenting both events. The cumulative incidence of CHD during follow-up was 21% (n = 55), and its incidence rate was 1.8 per 100 person-years. The incidence of CHD by tertiles of LTL distribution was 28% (T1), 22% (T2) and 13% (T3), respectively (log-rank p = 0.03, Fig. 1). Cox analyses confirmed the association of short LTL at baseline with increased risk of CHD during follow-up (Table 2): HR 2.55 (95% CI 1.26–5.57), for T1 vs T3 LTL, p = 0.009. The association remained significant following adjustments for age and traditional CHD risk factors (Model 1) and following further adjustment for history of myocardial infarction at baseline (Model 2). Associations were also observed for LTL expressed as a continuous variable. In a sensitivity analysis, LTL was also associated with new cases of CHD during follow-up when considering only participants without a history of CHD at baseline: HR 1.82 (95% CI 1.13–3.09), for T1 vs T3, p = 0.01 (Model 1).

The cumulative incidence of all-cause death during follow-up was 40% (n = 103), and its incidence rate was 2.7 per 100 person-years. All-cause death incidence by LTL tertiles was 51% (T1), 33% (T2) and 37% (T3), log-rank p = 0.03, Fig. 1. Given the similar incidences of all-cause death for T2 and T3 tertiles, their data were combined for the analyses of mortality. Cox analyses confirmed the association of short LTL at baseline with increased all-cause mortality risk during follow-up: HR 1.63 (95% CI 1.10–2.40) for T1 vs combined T2/T3 LTL, p = 0.02. The association remained significant following adjustments for traditional risk factors: HR 1.63 (95% CI 1.04–2.55) for T1 vs combined T2/T3 LTL, p = 0.03, adjusted Model 1. In competitive risk analyses considering all-cause death during follow-up as a competing risk, the LTL association with incident CHD remained significant in unadjusted and adjusted models (Additional file 1: Table S5).

### Risk of CHD during follow-up by allelic variations in telomere length related genes

The mean ± SD duration of follow-up for the 767 GENEDIAB or GENESIS participants selected for the SNP studies was 15 ± 6 years. Myocardial infarction and coronary revascularization occurred in, respectively, 86 (11%) and 74 (10%) participants during follow-up, with 41 participants presenting both events. The cumulative incidence of CHD during follow-up was 16% (n = 119), and its incidence rate was 1.4 per 100 person-years. Baseline characteristics of participants by the incidence of CHD during follow-up are summarized in Table 1. Associations with the incidence of CHD were observed for SNPs in six out of the eleven loci that were studied: TERC, NAF1, TERT, TNKS, MEN1 and BICD1 (Table 3).

**Table 3:** Risk of CHD during follow-up in GENEDIAB and GENESIS cohorts by allelic variations in LTL-related genes

Gene	SNP	Genetic model and risk allele	Hazard ratio (95% CI)	p
TERC	rs12696304	Recessive G	2.37 (1.34–5.01)	0.007
TERC	rs2293607	Recessive C	2.98 (1.30–5.88)	0.01
TERC	rs1317082	Recessive G	2.76 (1.21–5.49)	0.02
TERC	rs10936601	Recessive T	2.77 (1.32–5.23)	0.009
TERC	rs16847897	Dominant G	1.16 (0.52–3.31)	0.77
NAF1	rs7675998	Dominant T	1.57 (1.04–2.39)	0.03
NAF1	rs6823843	Recessive T	2.05 (1.30–3.33)	0.002
TERT	rs7726159	Codominant C	1.39 (1.03–1.90)	0.03
TERT	rs2736098	Codominant A	1.07 (0.74–1.52)	0.72
TERT	rs401681	Dominant T	1.32 (0.84–2.19)	0.24
TNKS	rs11991621	Dominant C	1.73 (0.63–7.13)	0.32
TNKS	rs12549064	Recessive A	1.24 (0.79–1.99)	0.35
TNKS	rs10903314	Dominant C	3.60 (1.32–14.83)	0.009
TNKS	rs6990300	Dominant G	1.23 (0.82–1.85)	0.32
TNKS	rs11249943	Codominant A	1.21 (0.84–1.78)	0.31
TNKS	rs17150478	Codominant A	1.04 (0.72–1.53)	0.85
TERF1	rs2981084	Dominant A	1.24 (0.67–2.57)	0.52
STN1	rs10786775	Dominant G	1.15 (0.70– 1.83)	0.56
STN1	rs2487999	Dominant T	1.30 (0.80–2.05	0.28
STN1	rs9420907	Dominant C	1.04 (0.67–1.57)	0.87
STN1	rs11591710	Codominant C	1.01 (0.43–2.23)	0.99
MEN1	rs669976	Codominant T	1.91 (1.11–13.53)	0.02
MEN1	rs524386	Codominant T	1.68 (0.93–3.40)	0.09
MEN1	rs2957154	Codominant C	1.66 (1.09–2.54)	0.02
MRE11	rs12270338	Codominant C	1.12 (0.80–1.60)	0.51
MRE11	rs13447720	Recessive C	1.34 (0.55–2.78)	0.49
BICD1	rs2630578	Codominant C	1.55 (1.04–2.27)	0.03
BICD1	rs2125173	Dominant G	1.81 (1.09–2.92)	0.02
BICD1	rs10506083	Dominant A	1.24 (0.72–2.32)	0.45
BICD1	rs10844149	Dominant A	1.39 (0.94–2.08)	0.10
BICD1	rs1151026	Recessive G	1.94 (0.58–4.76)	0.25
MPHOSPH06	rs2967374	Codominant A	1.02 (0.71–1.45)	0.89
ZNF208	rs8105767	Dominant G	1.52 (0.93–2.39)	0.09

Hazard Ratio computed by Cox proportional hazards survival regression analysis for the risk allele of SNPs in pooled GENEDIAD/GENESIS cohorts. Adjusted for cohort membership, sex, age, BMI, duration of diabetes, HbA1c, eGFR, UAC, tobacco smoking, and use of ACE-Inhibitors, antihypertensive and lipid lowering drugs at baseline

*BICD1* Bicaudal D homolog 1 *MEN1* Multiple Endocrine Neoplasia type 1 *MPHOSPH6* M-phase Phosphoprotein 6 *MRE11A* Meiotic Recombination 11 homolog A *NAF1* Nuclear Assembly Factor 1 *STN1* STN1 Subunit of CST Complex *TERC* Telomerase RNA Component *TERF1* Telomeric repeat-binding factor 1 *TERT* Telomerase Reverse Transcriptase *TNKS* Tankyrase *ZNF208*: Zinc Finger Protein 208

## Discussion

In the present investigation, we observed associations of telomere shortening at baseline with the prevalence of myocardial infarction at baseline and with the incidence of CHD and all-cause death during a long-term follow-up, 12–15 years on average, in patients with long-standing type 1 diabetes. The associations were independent of sex, age, duration of diabetes, and a range of relevant confounding factors at baseline. The association with CHD incidence remained significant when treating all-cause death as a competing risk. Moreover, allelic variations in six genes related to telomere biology were also associated with CHD. As far as we know, this is the first report of independent and reliable associations between telomere shortening and CHD and all-cause mortality in patients with type 1 diabetes.

Associations between LTL and cardiovascular disease (CVD) or cardiovascular death were previously reported in the general population [8, 26, 28, 29], including a meta-analysis of 24 studies and ~ 44,000 participants [30], and also in people with type 2 diabetes [10]. Clinical investigations supported these epidemiological results. In a study in apparently healthy middle-aged individuals, LTL was inversely related to carotid intima-media thickness (IMT), a marker of cardiovascular risk, was shorter in individuals with asymptomatic plaques, and even more so in a group of patients with symptomatic CHD [31]. Increased phagocytic NADPH oxidase-dependent superoxide production and serum 8-OHdG levels (a marker of DNA oxidation) were observed in participants with shorter LTL and increased carotid IMT [31]. Increased oxidative stress and telomere shortening have been observed in endothelial and vascular smooth cells from aortic aneurysms [32].

The physiological and pathophysiological mechanisms leading to telomere-shortening are complex and only partially understood. They involve a number of genetic, epigenetic, environmental and pathological disorders, notably oxidative stress-mediated damage and inflammation [33]. Telomere shortening and CVD share many common risk factors, including tobacco smoking, alcohol consumption, obesity, arterial hypertension, diabetes mellitus, dyslipidemia, disrupted circadian rhythm, oxidative stress and chronic inflammation [6, 10, 34, 35]. In addition to sharing risk factors, an increasing body of data suggests that telomere shortening plays a direct role in the development of atherosclerosis and CVD. In a study of patients with unstable angina or acute coronary syndrome, shorter LTL was associated with high-risk plaque morphology on virtual histology intravascular ultrasound [36]. Monocytes with disrupted telomeres showed increased secretion of chemoattractant protein-1, IL-6, and IL-1beta and increased oxidative burst, suggesting that telomere shortening promotes high-risk plaque subtypes by increasing proinflammatory activity [36]. Recent data has focused on a novel pathophysiological mechanisms linking telomere shortening to CVD via clonal hematopoiesis of indeterminate potential (CHIP) [34]. With aging and telomere shortening, hematopoietic stem cells develop CHIP that further aggravates chronic inflammation through multiple CHIP-related mutant gene signaling pathways. Mutant clonally-derived cells, including macrophages, mast cells, and T cells, further increase chronic inflammation, cardiac remodeling and the risk of atherosclerosis, CHD, aortic stenosis, peripheral arterial occlusive diseases and heart failure [34]. Moreover, medications with beneficial effects on prevention and treatment of CVD, including statins, ACE-inhibitors and pioglitazone, were shown to enhance telomerase activity and reduce telomere attrition in vitro and in animal models, suggesting that telomeres might be therapeutic targets in heart diseases [15].

The six genes with allelic variations associated with CHD in the present investigation (TERT, TERC, TNKS, MEN1, NAF1 and BICD1) play a major role in telomere biology as shown in different studies discussed below. *TERT* encodes the telomerase reverse transcriptase, a catalytic subunit of the enzyme telomerase. The rs7726159 in intron 3 of *TERT* was shown to affect telomerase transcription level by modulating the interaction of the transcriptional factor MYC with *TERT*. The C-allele, associated with CHD in our study, was previously associated with lower telomerase activity [37], and with shorter LTL and with ischemic heart disease in the general population [26]. Other *TERT* variants were associated with CVD in the Women's Genome Health Study [38]. *TERC* encodes the telomerase RNA component, a non-coding RNA that together with TERT comprises the main unit of the telomerase complex. TERC serves as a template for TERT for the insertion of the repetitive G-rich DNA sequence to the ends of chromosomes. Associations of *TERC* variants with LTL [22], and with hypertension and CHD were reported in the general population [39, 40]. *TNKS* encodes the enzyme tankirase that promotes telomere elongation by interacting with the telomere repeat factor 1 (TERF1), a critical inhibitor of elongation. Poly-ADP-ribosylation of TERF1 by tankirase leads to the release of TERF1 from telomeres and allows telomerase to access telomeric DNA [41]. *TNKS* variants were associated with CVD in the Women's Genome Health Study [38]. *MEN1* encodes menin, a putative tumor suppressor associated with multiple endocrine neoplasia. Menin overexpression downregulates TERT and telomerase activity [42]. *MEN1* variants were previously associated with LTL in controls from the PLCO Cancer Screening Trial and the Nurses’ Health Study [25]. *NAF1* encodes the nuclear assembly factor 1 ribonucleoprotein (NAF-1), a protein located in the endoplasmic reticulum and mitochondrial membranes. NAF-1 is involved in telomerase maintenance by regulating the H/ACA box motif of TERC, a domain required for its stability and assembly into the mature telomerase complex. Rare variants in *NAF1* were shown to cosegregate with decreased expression of NAF-1, low TERC levels, short telomere length and a phenotype of familial pulmonary fibrosis and emphysema [43]. *BICD1* codes for the bicaudal D homolog 1, a protein involved in vacuolar traffic that seems to regulate telomere length by effects on the telomerase pathway [24]. The C-allele of rs2630578, associated with CHD in our study, was shown to disrupt a putative motif for the NF-Y transcription factor in the *BICD1* regulatory region, and to be associated with lower *BICD1* mRNA levels in leukocytes and shorter LTL [24]. It was also associated with decreased left ventricular function in people with hypertension and CHD [44].

Our work has several strengths including the investigation of binational and multicentric cohorts of patients with long-standing type 1 diabetes, 39 year-duration on average at the end of follow-up, with a good retention over a ~ 15-year follow-up. Also, participants at baseline were in their forties, an age at which premature mortality is unlikely to affect representativeness at baseline in the case of a frequent gene variant distribution. Indeed, Hardy–Weinberg equilibrium of genotypes was verified for all outcome-related subsets of participants. The cohorts were designed to investigate biomarkers and genetic determinants of vascular complications of diabetes, had comprehensive clinical and biological data at baseline and pre-specified vascular outcomes during follow-up. Clinical events in relation with diabetes, including premature death, could be observed in a sizeable proportion of participants during the lengthy follow-up.

First, due to missing data, only 70% of the cohorts' original participants were included in the present investigation. Clinical characteristics at baseline were similar in GENEDIAB participants selected or not for the LTL studies. However, in the pooled cohorts, participants not selected as compared to those selected for the SNP studies had lower BMI and eGFR, and more frequently a history of previous myocardial infarction at baseline. We cannot exclude that these clinical differences, especially regarding myocardial infarction at baseline, induced selection bias in our results. However, given the higher prevalence of myocardial infarction in subjects not selected for the investigation, we would expect these bias to result in lack of power and/or false negative associations in the subset remaining in the study. Second, due to the relatively small population size, we had to use a composite outcome to ensure a minimal number of CHD events during follow-up. Acute myocardial infarction and elective coronary arteries revascularization (mostly for chronic angina or silent myocardial ischemia) may be heterogeneous regarding telomere shortening implication. However, it is noteworthy that our sample size is within the range of those of several studies showing an association of LTL with cardiovascular disease in the general population [8]. Third, our study did not have sufficient power to assess associations between SNPs and LTL, and thus we were not able to apply mendelian randomization to argue for the causality between LTL and CHD. However, these SNPs were previously associated with LTL in general population [13, 22–26], which is in agreement with our findings of LTL and SNPs associations with CHD. Fourth, we have chosen not to adjust for multiple comparisons the statistical significance of SNPs analyses. Even considering the linkage disequilibrium between SNPs located in same gene region, given the population size and small number of events, our study did not have sufficient statistical power to allow for such adjustments. We considered our results to be replications in type 1 diabetes cohorts of results from GWAS or candidate gene studies in the general population. Finally, we studied two cohorts consisting predominantly of people of European descent and the conclusion of SNP studies may not apply to people from other ethnic backgrounds.

## Conclusions

In conclusion, we reported independent and consistent association between relative LTL shortening and excess risk of CHD in patients with long-standing type 1 diabetes. These results were supported by associations with CHD in our cohorts of allelic variations in telomere biology-related genes associated with telomere shortening in the general population. The sensitivity and specificity of LTL as a marker of CVD, as well as its added value in relation to traditional cardiovascular risk factors still need to be established in patients with diabetes as well as in the general population. Environmental, pharmacological and heritable factors, as well as behavioral and lifestyle factors, affect telomere length throughout lifetime. Further studies in larger cohorts with dynamic assessment of telomere shortening through multiple LTL measures throughout follow-up and with enough statistical power to assess interactions with lifetime determinants of LTL should provide a much better view of the role of telomere shortening as a risk factor and as a biomarker of CVD. Further investigations are also needed to elucidate the pathophysiological mechanisms behind the association of telomere shortening and CVD, but also to assess the impact of modifiable factors on telomere length, and their relevance in treatment and prevention of CVD in people with diabetes.

## Supplementary Information

**Additional file 1: Table S1.** Baseline characteristics of GENEDIAB and GENESIS participants included in the present investigation. **Table S2.** Baseline characteristics of participants included or not in the LTL or the SNP studies. **Table S3.** SNPs in LTL-related genes genotyped in GENEDIAB and GENESIS participants. **Table S4.** Baseline characteristics of GENEDIAB participant by LTL tertiles of LTL distribution. **Table S5.** CHD risk during follow-up in the GENEDIAB cohort by baseline LTL with all-cause death as a competing risk. **Figure S1.** Flow chart of participants.

## Data Availability

The datasets used and/or analyzed during the current study are available from the corresponding author on reasonable request.

## Abbreviations

BICD1: Bicaudal D homolog 1
BMI: Body Mass Index
CHD: Coronary Heart Disease
CI: Confident Interval
CVD: Cardiovascular Disease
eGFR: Estimated Glomerular Filtration Rate
GAPDH: Glyceraldehyde-3-Phosphate-Deshydrogenase
GWAS: Genome-Wide Association Studies
HbA1c: Glycated Hemoglobin
HR: Hazard Ratio
IQR: Interquartile Range
LTL: Leukocyte Telomere Length
MEN1: Multiple Endocrine Neoplasia type 1
MPHOSPH6: M-phase Phosphoprotein 6
MRE11A: Meiotic Recombination 11 homolog A
NAF1: Nuclear Assembly Factor 1
OR: Odds Ratio
PCR: Polymerase Chain Reaction
SNP: Single Nucleotide Polymorphism
STN1: STN1 Subunit of CST Complex
TERC: Telomerase RNA Component
TERF1: Telomeric repeat-binding factor 1
TERT: Telomerase Reverse Transcriptase
TNKS: Tankyrase
UAC: Urinary Albumin Concentration
ZNF208: Zinc Finger Protein 208

## References

[1] Beckman JA, Creager MA, Libby P (2002). Diabetes and atherosclerosis: epidemiology, pathophysiology, and management. JAMA.

[2] Diabetes Control and Complications Trial (DCCT) /Epidemiology of Diabetes Interventions and Complications (EDIC) Study Research Group (2016). Intensive diabetes treatment and cardiovascular outcomes in type 1 diabetes: The DCCT/EDIC study 30-year follow-up. Diabetes Care.

[3] Lind M, Svensson A-M, Kosiborod M, Gudbjörnsdottir S, Pivodic A, Wedel H, Dahlqvist S, Clements M, Rosengren A (2014). Glycemic control and excess mortality in type 1 diabetes. N Engl J Med.

[4] Blackburn EH (1991). Structure and function of telomeres. Nature.

[5] Campisi J, Robert L (2014). Cell senescence: role in aging and age-related diseases. Interdiscip Top Gerontol.

[6] Minamino T, Komuro I (2008). Role of telomeres in vascular senescence. Front Biosci.

[7] Willeit P, Willeit J, Brandstätter A, Ehrlenbach S, Mayr A, Gasperi A, Weger S, Oberhollenzer F, Reindl M, Kronenberg F, Kiechl S (2010). Cellular aging reflected by leukocyte telomere length predicts advanced atherosclerosis and cardiovascular disease risk. Arterioscler Thromb Vasc Biol.

[8] Haycock PC, Heydon EE, Kaptoge S, Butterworth AS, Thompson A, Willeit P (2014). Leucocyte telomere length and risk of cardiovascular disease: systematic review and meta-analysis. BMJ.

[9] Donato AJ, Morgan RG, Walker AE, Lesniewski LA (2015). Cellular and molecular biology of aging endothelial cells. J Mol Cell Cardiol.

[10] Masi S, D'Aiuto F, Cooper J, Salpea K, Stephens JW, Hurel SJ, Deanfield JE, Humphries SE (2016). Telomere length, antioxidant status and incidence of ischaemic heart disease in type 2 diabetes. Int J Cardiol.

[11] Fyhrquist F, Tiitu A, Saijonmaa O, Forsblom C, Groop P-H (2010). Telomere length and progression of diabetic nephropathy in patients with type 1 diabetes. J Intern Med.

[12] Sanchez M, Hoang S, Kannengiesser C, Potier L, Hadjadj S, Marre M, Roussel R, Velho G, Mohammedi K (2020). Leukocyte telomere length, DNA oxidation, and risk of lower-extremity amputation in patients with long-standing type 1 diabetes. Diabetes Care.

[13] Codd V, Nelson CP, Albrecht E, Mangino M, Deelen J, Buxton JL, Hottenga JJ, Fischer K, Esko T, Surakka I, Broer L, Nyholt DR, Mateo Leach I, Salo P, Hägg S, Matthews MK, Palmen J, Norata GD, O'Reilly PF, Saleheen D, Amin N, Balmforth AJ, Beekman M, de Boer RA, Böhringer S, Braund PS, Burton PR, de Craen AJM, Denniff M, Dong Y, Douroudis K, Dubinina E, Eriksson JG, Garlaschelli K, Guo D, Hartikainen A-L, Henders AK, Houwing-Duistermaat JJ, Kananen L, Karssen LC, Kettunen J, Klopp N, Lagou V, van Leeuwen EM, Madden PA, Mägi R, Magnusson PKE, Männistö S, McCarthy MI, Medland SE, Mihailov E, Montgomery GW, Oostra BA, Palotie A, Peters A, Pollard H, Pouta A, Prokopenko I, Ripatti S, Salomaa V, Suchiman HED, Valdes AM, Verweij N, Viñuela A, Wang X, Wichmann H-E, Widen E, Willemsen G, Wright MJ, Xia K, Xiao X, van Veldhuisen DJ, Catapano AL, Tobin MD, Hall AS, Blakemore AIF, van Gilst WH, Zhu H, Erdmann J, Reilly MP, Kathiresan S, Schunkert H, Talmud PJ, Pedersen NL, Perola M, Ouwehand W, Kaprio J, Martin NG, van Duijn CM, Hovatta I, Gieger C, Metspalu A, Boomsma DI, Jarvelin M-R, Slagboom PE, Thompson JR, Spector TD, van der Harst P, Samani NJ, CARDIoGRAM consortium (2013). Identification of seven loci affecting mean telomere length and their association with disease. Nat Genetics..

[14] Valdes AM, Andrew T, Gardner JP, Kimura M, Oelsner E, Cherkas LF, Aviv A, Spector TD (2005). Obesity, cigarette smoking, and telomere length in women. Lancet.

[15] Yeh JK, Lin MH, Wang CY (2019). Telomeres as therapeutic targets in heart disease. JACC Basic Transl Sci.

[16] Marre M, Jeunemaitre X, Gallois Y, Rodier M, Chatellier G, Sert C, Dusselier L, Kahal Z, Chaillous L, Halimi S, Muller A, Sackmann H, Bauduceau B, Bled F, Passa P, Alhenc-Gelas F (1997). Contribution of genetic polymorphism in the renin-angiotensin system to the development of renal complications in insulin-dependent diabetes: Genetique de la Nephropathie Diabetique (GENEDIAB) study group. J Clin Invest.

[17] Hadjadj S, Pean F, Gallois Y, Passa P, Aubert R, Weekers L, Rigalleau V, Bauduceau B, Bekherraz A, Roussel R, Dussol B, Rodier M, Marechaud R, Lefebvre PJ, Marre M (2004). Different patterns of insulin resistance in relatives of type 1 diabetic patients with retinopathy or nephropathy: the Genesis France-Belgium Study. Diabetes Care.

[18] Cawthon RM (2002). Telomere measurement by quantitative PCR. Nucleic Acids Res.

[19] Cawthon RM (2009). Telomere length measurement by a novel monochrome multiplex quantitative PCR method. Nucleic Acids Res.

[20] Livak KJ, Schmittgen TD (2001). Analysis of relative gene expression data using real-time quantitative PCR and the 2(-Delta Delta C(T)) Method. Methods.

[21] Pfaffl MW, Filion M (2012). Quantification strategies in real-time RT–PCR (RT-qPCR). Quantitative real-time PCR in applied microbiology.

[22] Codd V, Mangino M, van der Harst P, Braund PS, Kaiser M, Beveridge AJ, Rafelt S, Moore J, Nelson C, Soranzo N, Zhai G, Valdes AM, Blackburn H, Mateo Leach I, de Boer RA, Kimura M, Aviv A, Goodall AH, Ouwehand W, van Veldhuisen DJ, van Gilst WH, Navis G, Burton PR, Tobin MD, Hall AS, Thompson JR, Spector T, Samani NJ (2010). Common variants near TERC are associated with mean telomere length. Nat Genet.

[23] Crocco P, Barale R, Rose G, Rizzato C, Santoro A, De Rango F, Carrai M, Fogar P, Monti D, Biondi F, Bucci L, Ostan R, Tallaro F, Montesanto A, Zambon CF, Franceschi C, Canzian F, Passarino G, Campa D (2015). Population-specific association of genes for telomere-associated proteins with longevity in an Italian population. Biogerontology.

[24] Mangino M, Brouilette S, Braund PS, Tirmizi N, Vasa-Nicotera M, Thompson JR, Samani NJ (2008). A regulatory SNP of the BICD1 gene contributes to telomere length variation in humans. Hum Mol Genet.

[25] Mirabello L, Yu K, Kraft P, De Vivo I, Hunter DJ, Prescott J, Wong JYY, Chatterjee N, Hayes RB, Savage SA (2010). The association of telomere length and genetic variation in telomere biology genes. Hum Mutat.

[26] Scheller Madrid A, Rode L, Nordestgaard BG, Bojesen SE (2016). Short Telomere length and ischemic heart disease: observational and genetic studies in 290,022 individuals. Clin Chem.

[27] Fine JP, Gray RJ (1999). A proportional hazards model for the subdistribution of a competing risk. J Am Stat Assoc.

[28] Brouilette SW, Moore JS, McMahon AD, Thompson JR, Ford I, Shepherd J, Packard CJ, Samani NJ, West of Scotland Coronary Prevention Study Group (2007). Telomere length, risk of coronary heart disease, and statin treatment in the West of Scotland primary prevention study: a nested case-control study. Lancet.

[29] Epel ES, Merkin SS, Cawthon R, Blackburn EH, Adler NE, Pletcher MJ, Seeman TE (2008). The rate of leukocyte telomere shortening predicts mortality from cardiovascular disease in elderly men. Aging (Albany NY).

[30] D'Mello MJJ, Ross SA, Briel M, Anand SS, Gerstein H, Paré G (2015). Association between shortened leukocyte telomere length and cardiometabolic outcomes: systematic review and meta-analysis. Circ Cardiovasc Genet.

[31] Pejenaute A, Cortes A, Marques J, Montero L, Beloqui O, Fortuno A, Marti A, Orbe J, Zalba G (2020). NADPH oxidase overactivity underlies telomere shortening in human atherosclerosis. Int J Mol Sci.

[32] Cafueri G, Parodi F, Pistorio A, Bertolotto M, Ventura F, Gambini C, Bianco P, Dallegri F, Pistoia V, Pezzolo A, Palombo D (2012). Endothelial and smooth muscle cells from abdominal aortic aneurysm have increased oxidative stress and telomere attrition. PLoS ONE.

[33] Blackburn EH, Epel ES, Lin J (2015). Human telomere biology: a contributory and interactive factor in aging, disease risks, and protection. Science.

[34] Huang YC, Wang CY (2021). Telomere attrition and clonal hematopoiesis of indeterminate potential in cardiovascular disease. Int J Mol Sci.

[35] McAlpine CS, Swirski FK (2016). Circadian influence on metabolism and inflammation in atherosclerosis. Circ Res.

[36] Calvert PA, Liew TV, Gorenne I, Clarke M, Costopoulos C, Obaid DR, O'Sullivan M, Shapiro LM, McNab DC, Densem CG, Schofield PM, Braganza D, Clarke SC, Ray KK, West NE, Bennett MR (2011). Leukocyte telomere length is associated with high-risk plaques on virtual histology intravascular ultrasound and increased proinflammatory activity. Arterioscler Thromb Vasc Biol.

[37] Li Y, Xiang C, Shen N, Deng L, Luo X, Yuan P, Ji Z, Li J, Cheng L (2019). Functional polymorphisms on chromosome 5p15.33 disturb telomere biology and confer the risk of non-small cell lung cancer in Chinese population. Mol Carcinog.

[38] Zee RY, Ridker PM, Chasman DI (2011). Genetic variants in eleven telomere-associated genes and the risk of incident cardio/cerebrovascular disease: the Women's Genome Health Study. Clin Chim Acta.

[39] Paik JK, Kang R, Cho Y, Shin M-J (2016). Association between genetic variations affecting mean telomere length and the prevalence of hypertension and coronary heart disease in Koreans. Clin Nutr Res.

[40] Perez-Rivera JA, Pabon-Osuna P, Cieza-Borrella C, Lugo-Godoy C, Martin-Herrero F, Gonzalez-Porras JR, Sanchez-Fernandez PL, Gonzalez-Sarmiento R (2015). The role of the TERC-63G>A and TERT-1327C>T telomerase polymorphisms in the study of men with acute coronary syndrome. Minerva Cardioangiol.

[41] Li B, Qiao R, Wang Z, Zhou W, Li X, Xu W, Rao Z (2016). Crystal structure of a tankyrase 1-telomere repeat factor 1 complex. Acta Crystallogr F Struct Biol Commun.

[42] Lin SY, Elledge SJ (2003). Multiple tumor suppressor pathways negatively regulate telomerase. Cell.

[43] Stanley SE, Gable DL, Wagner CL, Carlile TM, Hanumanthu VS, Podlevsky JD, Khalil SE, DeZern AE, Rojas-Duran MF, Applegate CD, Alder JK, Parry EM, Gilbert WV, Armanios M (2016). Loss-of-function mutations in the RNA biogenesis factor *NAF1* predispose to pulmonary fibrosis–emphysema. Sci Transl Med.

[44] Huber M, Treszl A, Wehland M, Winther I, Zergibel I, Reibis R, Bolbrinker J, Stoll M, Schonfelder G, Wegscheider K, Voller H, Kreutz R (2012). Genetic variants implicated in telomere length associated with left ventricular function in patients with hypertension and cardiac organ damage. J Mol Med (Berl).

