# Supplementary material for: Leukocyte telomere length, allelic variations in related genes and risk of coronary heart disease in people with long-standing type 1 diabetes
Source: Cardiovasc Diabetol. 2022 Oct 11;21:206. doi: 10.1186/s12933-022-01635-0 (PMC9554968; doi:10.1186/s12933-022-01635-0)
Supplement: Supplementary file 1 — Additional file 1: Table S1. Baseline characteristics of GENEDIAB and GENESIS participants included in the present investigation. Table S2. Baseline characteristics of participants included or not in the LTL or the SNP studies. Table S3. SNPs in LTL-related genes genotyped in GENEDIAB and GENESIS participants. Table S4. Baseline characteristics of GENEDIAB participant by LTL tertiles of LTL distribution. Table S5. CHD risk during follow-up in the GENEDIAB cohort by baseline LTL with all-cause death as a competing risk. Figure S1. Flow chart of participants. [file 12933_2022_1635_MOESM1_ESM.docx]

**Leukocyte telomere length, related allelic variations and risk of coronary events in people with long-standing type 1 diabetes**

**Supplemental Material**

Page 2. Supplemental Table 1. Baseline characteristics of GENEDIAB and GENESIS participants included in the present investigation

Page 3. Supplemental Table 2. Baseline characteristics of participants included or not in the LTL or SNP studies

Page 5. Supplemental Table 3. SNPs in LTL-related genes genotyped in GENEDIAB and GENESIS participants

Page 7. Supplemental Table 4. Baseline characteristics of GENEDIAB participant by tertiles of LTL distribution

Page 9. Supplemental Table 5. CHD risk during follow-up in the GENEDIAB cohort by baseline LTL, with all-cause death as a competing risk

Page 10 Supplemental Figure 1. Flow chart of participants

Page 11 List of Contributors to GENEDIAB and GENESIS studies

**Table S1. Baseline characteristics of GENEDIAB and GENESIS participants included in the present investigation**

|  | **GENEDIAB** | **GENESIS** | **p** |
| --- | --- | --- | --- |
| N | 323 | 444 |  |
| Sex: male, n (%) | 192 (59) | 229 (52) | 0.03 |
| Age, y | 45 ± 12 | 42 ± 11 | <0.0001 |
| Duration of diabetes, y | 29 ± 9 | 26 ± 9 | 0.0008 |
| BMI, kg/m^2^ | 24.0 ± 3.3 | 24.4 ± 3.6 | 0.13 |
| Systolic blood pressure, mmHg | 139 ± 18 | 131 ± 20 | <0.0001 |
| Diastolic blood pressure, mmHg | 79 ± 12 | 76 ± 10 | <0.0001 |
| HbA1c, % | 8.6 ± 1.6 | 8.5 ± 1.4 | 0.69 |
| HbA1c, mmol/mol | 70 ± 18 | 70 ± 15 | 0.69 |
| Total cholesterol, mmol/l | 5.71 ± 1.44 | - | - |
| eGFR, ml/min/1.73 m^2^ | 74 ± 29 | 88 ± 32 | <0.0001 |
| UAC, mg/l* | 33 [453] | 20 [145] | 0.09 |
| Current tobacco smoking, n (%) | 79 (25) | 133 (30) | 0.12 |
| Previous myocardial infarction, n (%) | 20 (6) | 10 (2) | 0.008 |
| Previous stroke, n (%) | 12 (4) | 9 (2) | 0.18 |
| Previous LLA, n (%) | 57 (18) | 7 (2) | <0.0001 |
| Use of lipid lowering drugs, n (%) | 32 (10) | 29 (7) | 0.10 |
| Use of blood pressure lowering drugs, n (%) | 189 (57) | 216 (51) | 0.005 |
| Use of ACE-I, n (%) | 147 (46) | 165 (37) | 0.02 |

Quantitative data expressed as mean ± SD or median [IQR]*. Data from GENEDIAB represent participants included in the SNP studies (n=323). Of these, 260 participants were also included in the LTL studies. Total cholesterol levels available only for GENEDIAB participants. Statistics are Student's t test, *Kruskal-Wallis test or Fisher's exact test. eGFR, estimated glomerular filtration rate. UAC: urinary albumin concentration. LLA: lower limb amputation. ACE-I: angiotensin converting enzyme inhibitor. p<0.05 was significant.

**Table S2. Baseline characteristics of participants included or not in the LTL or the SNP studies**

|  | **LTL studies (GENEDIAB cohort)** | | |  | **SNP studies (pooled cohorts)** | | |
| --- | --- | --- | --- | --- | --- | --- | --- |
|  | **Study inclusion** | | **p** |  | **Study inclusion** | | **p** |
|  | **No** | **Yes** |  |  | **No** | **Yes** |  |
| N | 259 | 260 |  |  | 333 | 767 |  |
| Sex: male, n (%) | 136 (53) | 155 (60) | 0.10 |  | 168 (50) | 421 (55) | 0.19 |
| Age, y | 44 ± 12 | 45 ± 12 | 0.86 |  | 44 ± 13 | 43 ± 11 | 0.11 |
| Duration of diabetes, y | 29 ± 10 | 29 ± 10 | 0.78 |  | 28 ± 10 | 27 ± 9 | 0.81 |
| BMI, kg/m^2^ | 23.5 ± 3.2 | 24.0 ± 3.3 | 0.07 |  | 23.7 ± 3.4 | 24.3 ± 3.5 | 0.01 |
| Systolic blood pressure, mmHg | 137 ± 19 | 139 ± 18 | 0.19 |  | 134 ± 19 | 135 ± 19 | 0.79 |
| Diastolic blood pressure, mmHg | 79 ± 11 | 79 ± 12 | 0.51 |  | 77 ± 11 | 77 ± 11 | 0.90 |
| HbA1c, % | 8.6 ± 1.9 | 8.6 ± 1.6 | 0.89 |  | 8.5 ± 1.6 | 8.5 ± 1.4 | 0.73 |
| HbA1c, mmol/mol | 70 ± 21 | 70 ± 17 | 0.89 |  | 70 ± 18 | 70 ± 16 | 0.73 |
| Total cholesterol, mmol/l | 5.73 ± 1.52 | 5.66 ± 1.35 | 0.63 |  | - | - | - |
| eGFR, ml/min/1.73 m^2^ | 70 ± 29 | 74 ± 30 | 0.08 |  | 73 ± 30 | 82 ± 32 | <0.0001 |
| UAC, mg/l* | 33 [511] | 36 [450] | 0.83 |  | 27 [359] | 23 [225] | 0.77 |
| Current tobacco smoking, n (%) | 72 (28) | 62 (24) | 0.25 |  | 102 (31) | 212 (28) | 0.31 |
| Previous myocardial infarction, n (%) | 20 (8) | 18 (7) | 0.73 |  | 33 (10) | 30 (4) | 0.0002 |
| Previous stroke, n (%) | 12 (5) | 9 (3) | 0.48 |  | 15 (5) | 21 (3) | 0.14 |
| Previous LLA, n (%) | 40 (16) | 48 (19) | 0.51 |  | 35 (12) | 64 (9) | 0.13 |
| Use of lipid lowering drugs, n (%) | 26 (10) | 21 (8) | 0.43 |  | 33 (10) | 61 (8) | 0.29 |
| Use of blood pressure lowering drugs, n (%) | 142 (57) | 148 (58) | 0.86 |  | 168 (52) | 505 (53) | 0.74 |
| Use of ACE-I, n (%) | 105 (43) | 116 (45) | 0.61 |  | 117 (37) | 312 (41) | 0.22 |

Quantitative data expressed as mean ± SD or median [IQR]*. Total cholesterol levels available only for GENEDIAB participants. Statistics are Student's t test, *Kruskal-Wallis test or Fisher's exact test. eGFR, estimated glomerular filtration rate. UAC: urinary albumin concentration. LLA: lower limb amputation. ACE-I: angiotensin converting enzyme inhibitor. p<0.05 was significant.

**Table S3: SNPs in LTL-related genes genotyped in GENEDIAB and GENESIS participants**

| Gene | Chromosome | SNP | Minor allele | MAF | Genotyping success rate (%) | P for HWE | Reference |
| --- | --- | --- | --- | --- | --- | --- | --- |
| TERC | 3q26.2 | rs12696304 | G | 0.26 | 96 | 0.62 | (20) |
|  |  | rs2293607 | C | 0.22 | 90 | 0.77 |  |
|  |  | rs1317082 | G | 0.22 | 92 | 0.92 |  |
|  |  | rs10936601 | T | 0.25 | 89 | 0.79 |  |
|  |  | rs16847897 | C | 0.27 | 92 | 0.52 |  |
| NAF1 | 4q32.2 | rs7675998 | A | 0.23 | 89 | 0.47 | (13) |
|  |  | rs6823843 | C | 0.24 | 85 | 0.20 |  |
| TERT | 5p15.33 | rs7726159 | A | 0.38 | 88 | 0.02 | (13,24) |
|  |  | rs2736098 | T | 0.24 | 83 | 0.06 |  |
|  |  | rs401681 | T | 0.47 | 94 | 0.32 |  |
| TNKS | 8p23.1 | rs11991621 | T | 0.18 | 92 | 0.17 | (23) |
|  |  | rs12549064 | C | 0.17 | 90 | 0.89 |  |
|  |  | rs10903314 | T | 0.26 | 94 | 0.05 |  |
|  |  | rs6990300 | G | 0.34 | 93 | 0.70 |  |
|  |  | rs11249943 | C | 0.19 | 92 | 0.57 |  |
|  |  | rs17150478 | G | 0.18 | 89 | 0.60 |  |
| TERF1 | 8q21.11 | rs2981084 | G | 0.31 | 93 | 0.73 | (21) |
| STN1 | 10q24.33 | rs10786775 | G | 0.09 | 94 | 0.58 | (13) |
|  |  | rs2487999 | T | 0.10 | 88 | 0.27 |  |
|  |  | rs9420907 | C | 0.15 | 91 | 0.05 |  |
|  |  | rs11591710 | C | 0.13 | 93 | 0.03 |  |
| MEN1 | 11q13 | rs669976 | C | 0.10 | 89 | 0.17 | (23) |
|  |  | rs524386 | C | 0.08 | 95 | 0.18 |  |
|  |  | rs2957154 | C | 0.25 | 92 | 0.39 |  |
| MRE11 | 11q21 | rs12270338 | A | 0.21 | 93 | 0.50 | (23) |
|  |  | rs13447720 | C | 0.22 | 93 | 0.58 |  |
| BICD1 | 12p11.21 | rs2630578 | C | 0.18 | 83 | 0.06 | (22) |
|  |  | rs2125173 | G | 0.11 | 91 | 0.41 |  |
|  |  | rs10506083 | G | 0.42 | 91 | 0.35 |  |
|  |  | rs10844149 | A | 0.28 | 96 | 0.75 |  |
|  |  | rs1151026 | G | 0.19 | 92 | 0.42 |  |
| MPHOSPH6 | 16q23.3 | rs2967374 | A | 0.21 | 89 | 0.37 | (13) |
| ZNF208 | 19p12 | rs8105767 | G | 0.10 | 95 | 0.55 | (13) |

SNPs were genotyped in 767 GENESIS or GENEDIAB participants. MAF: Minor allele frequency. HWE: Hardy-Weinberg equilibrium.

BICD1: Bicaudal D homolog 1. MEN1: Multiple Endocrine Neoplasia type 1. MPHOSPH6: M-phase Phosphoprotein 6. MRE11A: Meiotic Recombination 11 homolog A. NAF1: Nuclear Assembly Factor 1. STN1: STN1 Subunit of CST Complex. TERC: Telomerase RNA Component. TERF1: Telomeric repeat-binding factor 1. TERT: Telomerase Reverse Transcriptase. TNKS: Tankyrase. ZNF208: Zinc Finger Protein 208.

**Table S4. Baseline characteristics of GENEDIAB participant by LTL tertiles of LTL distribution**

|  | **T1** | **T2** | **T3** | **p** |
| --- | --- | --- | --- | --- |
| N | 90 | 92 | 78 | - |
| LTL, T/S ratio* | 0.80 [0.49] | 1.37 [0.40] | 2.50 [1.72] | <0.0001 |
| Sex: male, n (%) | 55 (61) | 59 (64) | 41 (53) | 0.29 |
| Age, years | 47 ± 12 | 44 ± 12 | 42 ± 12 | 0.04 |
| Duration of diabetes, years | 29 ± 9 | 29 ± 9 | 28 ± 11 | 0.80 |
| BMI, kg/m^2^ | 24.2 ± 3.6 | 24.1 ± 2.9 | 23.5 ± 3.3 | 0.34 |
| Systolic blood pressure, mmHg | 141 ± 18 | 138 ± 16 | 138 ± 21 | 0.62 |
| Diastolic blood pressure, mmHg | 80 ± 11 | 78 ± 11 | 80 ± 14 | 0.55 |
| HbA1c, % | 8.7 ± 1.7 | 8.4 ± 1.4 | 8.5 ± 1.6 | 0.37 |
| HbA1c, mmol/mol | 72 ± 18 | 68 ± 15 | 69 ± 18 | 0.37 |
| Total cholesterol, mmol/l | 5.61 ± 1.33 | 5.74 ± 1.31 | 5.59 ± 1.45 | 0.77 |
| eGFR, ml/min/1.73 m^2^ | 71 ± 29 | 76 ± 31 | 75 ± 30 | 0.51 |
| UAC, mg/l* | 53 [374] | 27 [454] | 46 [532] | 0.95 |

| UAC stages: Normoalbuminuria, n (%) | 32 (35) | 41 (45) | 24 (30) |  |
| --- | --- | --- | --- | --- |
| Microalbuminuria, n (%) | 15 (17) | 16 (17) | 22 (29) | 0.09 |
| Macroalbuminuria, n (%) | 43 (48) | 35 (38) | 32 (41) |  |
| Current tobacco smoking, n (%) | 26 (29) | 19 (21) | 17 (22) | 0.39 |
| Previous myocardial infarction, n (%) | 11 (12.2) | 4 (4.7) | 3 (3.6) | 0.04 |
| Previous stroke, n (%) | 3 (3.3) | 3 (3.5) | 3 (3.6) | 0.98 |
| Previous LLA, n (%) | 23 (26) | 16 (19) | 9 (11) | 0.04 |
| Use of lipid lowering drugs, n (%) | 6 (7) | 10 (11) | 5 (6) | 0.47 |
| Use of blood pressure lowering drugs, n (%) | 53 (59) | 50 (56) | 45 (58) | 0.93 |
| Use of ACE-I, n (%) | 38 (42) | 43 (48) | 35 (45) | 0.71 |

Quantitative data expressed as mean ± SD or median [IQR]*. Statistics are ANOVA, *Kruskal-Wallis test or Pearson's chi-squared test. Tertiles of LTL distribution at baseline: T1 (short LTL), T2 (intermediate LTL), T3 (long LTL). T/S ratio: Telomere to a single gene (used as a control) ratio (see methods). eGFR, estimated glomerular filtration rate. UAC: urinary albumin concentration. LLA: lower limb amputation. ACE-I: angiotensin converting enzyme inhibitor. p<0.05 was significant.

**Table S5. CHD risk during follow-up in the GENEDIAB cohort by baseline LTL with all-cause death as a competing risk**

|  |  | **Crude** | |  | **Adjusted Model 1** | |  | **Adjusted Model 2** | |
| --- | --- | --- | --- | --- | --- | --- | --- | --- | --- |
|  |  | **Subhazard Ratio (95% C.I.)** | **p** |  | **Subhazard Ratio (95% C.I.)** | **p** |  | **Subhazard Ratio (95% C.I.)** | **p** |
| T1 vs T3 |  | 2.40 (1.16 – 4.97) | 0.02 |  | 3.32 (1.34 – 8.23) | 0.009 |  | 3.23 (1.27 – 8.24) | 0.01 |
| T1 vs T2 |  | 1.34 (0.75 – 2.41) | 0.33 |  | 1.62 (0.77 – 3.41) | 0.20 |  | 2.47 (0.67 – 3.22) | 0.34 |
| T2 vs T3 |  | 1.79 (0.85 – 3.79) | 0.13 |  | 2.05 (0.76 – 5.53) | 0.16 |  | 2.20 (0.79 – 6.13) | 0.13 |
| Z-score log[LTL] |  | 0.78 (0.57 – 1.06) | 0.11 |  | 0.68 (0.50 – 0.94) | 0.02 |  | 0.71 (0.52 – 0.97) | 0.03 |

Subhazard Ratio computed by competing risk regression analysis for 1 SD of log[LTL] and for tertiles (T) of LTL distribution, with all-cause death during follow-up as a competing risk. T1 (short LTL), T2 (intermediate LTL), T3 (long LTL). Model 1: adjusted for sex, age, BMI, duration of diabetes, HbA1c, eGFR, UAC, tobacco smoking and use of ACE-Inhibitors, antihypertensive and lipid lowering drugs at baseline. Model 2: Model 1 plus adjustment for previous history of myocardial infarction at baseline. Number of participants with/without incident CHD during follow-up by LTL tertiles: 25/65 (T1), 20/72 (T2) and 10/68 (T3).

**Figure S1. Flow chart of participants**

**List of Contributors to GENEDIAB and GENESIS studies**

**Clinical investigators at baseline by alphabetical order of enrollment centers**

All cities are in France, except for Liège, in Belgium:

Albert Fournier, Jean-Daniel Lalau (*Centre Hospitalier Universitaire d’Amiens*); Béatrice Bouhanick, Line Godiveau, Michel Marre, Vincent Rohmer (*Centre Hospitalier Universitaire d’Angers*); Jean-Raymond Attali, Patrick Miossec (*Assistance Publique des Hôpitaux de Paris, Hôpital de Bondy*); Henri Gin, Vincent Rigalleau (*Centre Hospitalier Universitaire de Bordeaux*); Isabelle Cerf, Guillaume Charpentier, Isabelle Petit, Jean-Pierre Riveline (*Centre Hospitalier de Corbeil-Essonne*); Bertrand Godeau, Zoubida Kahal, Dominique Simon (*Assistance Publique des Hôpitaux de Paris, Hôpital de Créteil*); Daniel Cordonnier, Serge Halimi (*Centre Hospitalier Universitaire de Grenoble*); Pierre-Jean Lefebvre, Nicolas Paquot, André Scheen, Laurent Weekers (*Centre Hospitalier Universitaire de Liège*); Pierre Fontaine, Gaëtan Prevot (*Centre Hospitalier Régional Universitaire de Lille*); François Berthezene, Fabrice Bonnet, Maurice Laville, Jean-Pierre Fauvel, Charles Thivolet (*Assistance Publique des Hôpitaux de Lyon*); Bertrand Dussolle, Philippe Vague (*Assistance Publique des Hôpitaux de Marseille*); Patrick Giraud (*Clinique Pont de Chaume, Montauban*); Jacques Bringer, Florence Galtier, Michel Rodier (*Centres Hospitaliers Universitaires de Montpellier et Nîmes*); Pierre Drouin, Laurent Dusselier, Thérèse Crea, Bruno Guerci, Michèle Kessler (*Centre Hospitalier Universitaire de Nancy*); Lucy Chaillous, Bernard Charbonnel (*Centre Hospitalier Universitaire de Nantes*); Hamid Boukersi, Françoise Defrance, Etienne Larger, Michel Marre, Muriel Omani, Ronan Roussel (*Assistance Publique des Hôpitaux de Paris, Hôpital Bichat-Claude Bernard*); Gérard Slama, Agnès Sola (*Assistance Publique des Hôpitaux de Paris, Hôpital de l’Hôtel Dieu*); André Grimaldi, Agnès Heurtier, Caroline Sert (*Assistance Publique des Hôpitaux de Paris, Hôpital de La Pitié Salpétrière*); Jean-Pierre Grunfeld (*Assistance Publique des Hôpitaux de Paris, Hôpital Necker*); Ahmed Bouallouche, Jean-François Gautier, Pierre-Jean Guillausseau, Hervé Leblanc, Philippe Passa (*Assistance Publique des Hôpitaux de Paris, Hôpitaux Saint-Louis et Lariboisière*); Samy Hadjadj, Richard Maréchaud, Anne Muller, Florence Torremocha (*Centre Hospitalier Universitaire de Poitiers*); Jacques Chanard, Jean-Paul Melin (*Centre Hospitalier Universitaire de Reims*); Hubert Allannic, Jean-Yves Poirier (*Centre Hospitalier Universitaire de Rennes*); Christian Charasse (*Centre Hospitalier de Saint-Brieuc*); Bernard Bauduceau, Lyse Bordier, Hervé Mayaudon (*Hôpital d'Instruction des Armées Bégin, Saint-Mandé*); Marie-Pierre Arpin-Bott (*Centre Hospitalier Universitaire de Strasbour*g); Hélène Hanaire, Pierre Gourdy, Henri Sackmann, Jean-Pierre Tauber (*Centre Hospitalier Universitaire de Toulouse*); Odile Verier-Mine (*Centre Hospitalier de Valenciennes*).

**Associated laboratories**

Yves Gallois (*Laboratoire de Biochimie Universitaire, Centre Hospitalier Universitaire d’Angers*); François Alhenc-Gelas (*Centre de Recherche des Cordeliers, INSERM, Université de Paris, Sorbonne Université, Paris*).
